# Biological Sciences section of The Journals of Gerontology Series A

**DOI:** 10.1093/geroni/igaf122.1236

**Published:** 2025-12-31

**Authors:** Gustavo Duque

**Affiliations:** McGill University, Montreal, Quebec, Canada

## Abstract

The Biological Sciences section of The Journals of Gerontology Series A publishes articles on the biological aspects of aging in areas such as translational gerontology, biomarkers of aging, biochemistry, biodemography, cellular and molecular biology, comparative and evolutionary biology, endocrinology, exercise science, genetics, immunology, neuroscience, nutrition, pathology, pharmacology, physiology, and the biological mechanisms of late life diseases. Our primary focus is on mechanisms and novel insights in aging biology from diverse models such as cultured cells, invertebrates, vertebrates, and mammalian species including humans. The journal is particularly interested in publishing research on translational gerontology including interventions designed to enhance longevity and healthspan.

